# GAS2-like 1 coordinates cell division through its association with end-binding proteins

**DOI:** 10.1038/s41598-019-42242-6

**Published:** 2019-04-09

**Authors:** Alicja Nazgiewicz, Paul Atherton, Christoph Ballestrem

**Affiliations:** Wellcome Trust Centre for Cell-Matrix Research, Faculty of Biology, Medicine and Health, University of Manchester, Manchester, M13 9PT UK

## Abstract

Cell division involves the tightly coordinated rearrangement of actin and microtubules (MTs). We have previously shown that a member of the family of growth arrest-specific 2-like proteins, GAS2-like 1 (G2L1) regulates actin-MT crosstalk through its associations with plus-end microtubule tip-binding (EB) proteins. Here we show that G2L1 is involved in the regulation of cell division. We show that the depletion of G2L1 results in a reduction in the number of cells undergoing cell division and a significant proportion of those cells that do divide are either multinucleated, display deformed nuclei, or undergo cell division at a much slower rate. Exogenous expression of G2L1 mutants revealed that the association of G2L1 with EB1 is critical for regulated cell division and blocking this interaction inhibits cell division as observed in cells lacking G2L1. Taken together, our data suggest that G2L1 controls the precise regulation and successful progression of cell division through its binding to EB-proteins.

## Introduction

Cell division is a vital process in the lifetime of a cell. Any aberrations during this process can lead to severe health problems, and uncontrolled division is a key hallmark of cancer. Coordinated cell division requires precise rearrangements of the actin and microtubule (MT) cytoskeletal systems and the interplay between these two systems is vital. As cells round up prior to mitosis, actin stress fibres (SFs) disassemble into a cortical actomyosin network at the cell periphery. At the same time, MTs reorganise to form the mitotic spindle which is composed of^1^: kinetochore MTs linked to kinetochores on sister chromatids responsible for chromosome segregation^2^, non-kinetochore MTs that interact with the same MT type from the opposite spindle pole, and^3^ astral MTs that attach the spindle to the cell cortex. At the end of anaphase, dividing cells form an actin-based contractile ring, which forms the cleavage furrow during telophase. Components of the cleavage furrow are essential for separation into two daughter cells during cytokinesis (reviewed in)^4^.

A number of reports have shown that the synchronization of mitotic events requires the coordination of both actin and MT networks. For example, it was found that the cortical actin network plays an important role in spindle assembly, positioning, and length during mitosis^5–7^. The precise mechanism of how the actin-MT interplay is regulated at different stages of cell division remains to be elucidated.

**Figure 1:**
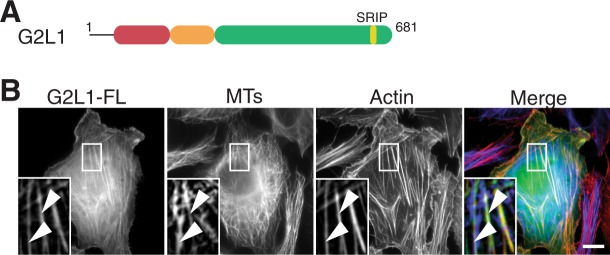
GAS2 family members and their subcellular localisation in U2OS cells. (**A**) Schematic representation of GAS2-Like1 (G2L1). The calponin homology (CH) and GAS2-related (GAR) domains are depicted in red and orange, respectively, and the number of amino acids is noted above the C-termini (green). The IP motives responsible for end-binding proteins (EB) binding are denoted above their respective C-termini, SxIP motif is yellow. (**B**) U2OS cell expressing GFP-G2L1. Cells were fixed and stained for MTs (blue) and actin (red). White arrowheads indicate co-aligning MTs and actin structures. Scale bar indicates 10 μm.

The GAS2 protein family consists of four members: the founding member of the family GAS2^8^, and three GAS2-like (G2L) proteins (GAS2-like 1 (G2L1), GAS2-like 2 (G2L2) and GAS2-like 3 (G2L3)), all of which have previously been shown to contribute to cytoskeletal regulation^9,10^. All members contain an actin-binding calponin homology (CH) domain and a putative MT-binding GAS2-related (GAR) domain. G2L1 and G2L2 contain a larger unstructured C-terminus domain with evolutionarily-conserved MT-tip localisation signals (MtLS) composed of the amino acid consensus sequence Ser/Thr-X-Ile/Leu-P (SxIP motifs) (Fig. 1A)^9^. This motif is required for the interaction with MT plus-end (+end)-binding (EB) proteins^11^ and regulate the crosstalk between MTs and F-actin^12,13^.

G2L proteins have previously been shown to contribute to the regulation of cell division. G2L3 knockout mice die early after birth because of cytokinesis defects^14^. Depletion of G2L3 resulted in defects in chromosome separation^15,16^, and overexpression of G2L3 specifically interferes with cell abscission at the final stage of cell division^17^. In addition to having a role in regulating cell motility^12^, G2L1 has also been reported to regulate centrosome splitting by mediating actin-microtubule crosstalk^1^. However, the precise role of G2L1 in cell division requires further elucidation.

EB proteins have a well-established role in cell division^18–21^. EB1 has been shown to be involved in anchoring MTs at the centrosome^22^, and has additional roles in regulating spindle positioning and symmetry^23,24^, the connection of MTs with kinetochores^18^ and cortical contractility^23^. Perturbation of EB1-MT association results in failure of chromosomal congression and delayed mitotic events^18^. EB3, on the other hand, has been shown to be responsible for stabilization of the midbody and focal adhesions (FA), which are required for coordinated spreading of daughter cells during cytokinesis^19^. Despite these observations it remains unclear how EB-proteins are engaged in these processes that are dependent on efficient cross-communication between actin and MTs.

Given the importance of both cytoskeletal systems in regulating mitotic events, and the strong regulatory link between G2L1 and EB proteins, we hypothesised a role for G2L1 in regulating cell division by mediating actin-MT crosstalk through EB proteins. In this study, we show that G2L1 localises to the mitotic spindle and cleavage furrow during mitosis and constriction zones of the midbody during late cytokinesis. We found that depletion of G2L1 leads to nuclear abnormalities and multinucleation therefore inhibiting and/or delaying cell division. These abnormalities and defects in cell division also occur upon expression of an EB-binding deficient mutant of G2L1. Our data leads to a model whereby G2L1 mediates actin-MT crosstalk through its binding to EB-proteins, to regulate the coordinated progression through cell division.

## Results

### G2L1 localises to both actin- and MT-related mitotic structures during cell division

Exogenous expression of G2L1 induces a strong co-alignment of MTs and actin stress fibres (SFs) in NIH 3T3 fibroblasts^13^. We aimed to confirm this finding in a human osteosarcoma cell line (U2OS) that is well established for cell division studies. As previously shown for fibroblasts, we detected a strong MT-actin co-alignment for cells exogenously expressing G2L1 (Fig. 1B).

**Figure 2:**
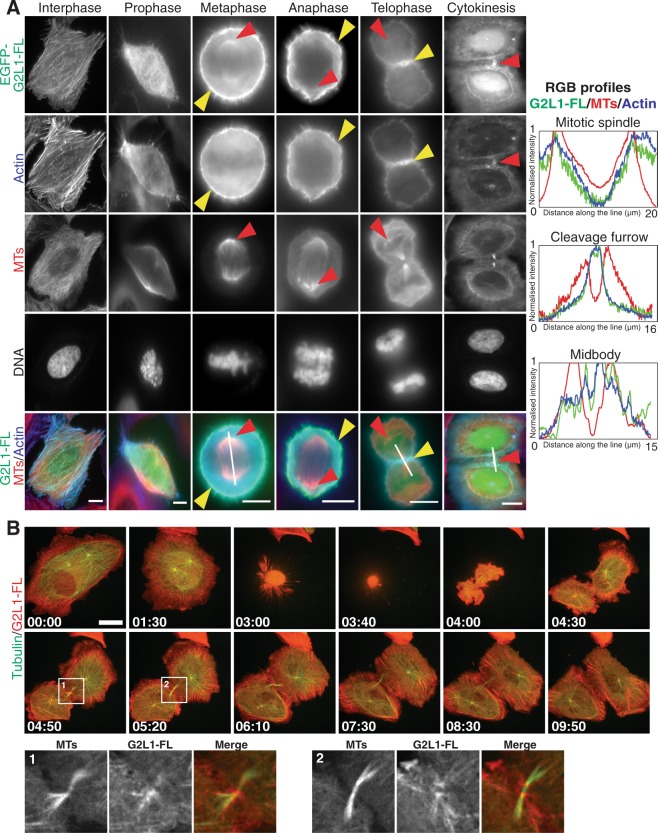
G2L1 accumulates at mitotic structures during cell division. (A) Panel of images represents U2OS cells expressing G2L1-FL (green) and stained for MTs (red), actin (blue) and DNA in different phases of cell division. Arrowheads indicate accumulation of G2L1-FL (red) at MT-related and (yellow) actin-related mitotic structures in dividing cells. White lines indicate region where measurements were taken for intensity line profiles, which are shown on the right. RGB profiles represent normalised intensity of G2L1 (green), MTs (blue) and actin (red). (**B**) Still time frames from a movie of a dividing cell expressing G2L1-FL (red) and MTs (green). Scale bars, 10 μm.

To investigate the potential role of G2L1 in cell division we expressed wild-type G2L1 (G2L1-FL-GFP) in U2OS cells and analysed its localisation at different phases of mitosis. To visualise its relation with the cytoskeletal systems we co-labelled cells for actin, MTs and DNA. Using this approach we identified G2L1 colocalisation with actin structures including the contractile ring, the cleavage furrow and constriction zones of the midbody. G2L1 also localised to MT-related structures such as the centrosome and the mitotic spindle during cell division (Fig. 2A,B, Supplemental Movie 1).

While exogenous G2L1-GFP localised to mitosis-related structures, it remained to be determined whether the endogenous protein shared this localisation. To detect endogenous G2L1 we used an antibody directed against the C-terminal tail of G2L1. To test specificity and recognition for G2L1, which has very low endogenous expression levels^16^, we firstly performed western blotting of cells expressing exogenous G2L1-FL-GFP. We found that the antibody detected GFP tagged G2L1 (Supplementary Fig. 1A). Similarly, immunofluorescence (IF) of cells expressing G2L1-FL-GFP using the same antibody demonstrated colocalisation with GFP tagged G2L1 indicating that the antibody was specific for G2L1 (Supplementary Fig. 1B).

We used this antibody to identify the localisation of endogenous G2L1 by IF in U2OS cells. We found faint localisation to MTs in cells in interphase, but observed an accumulation of G2L1 at constriction sites of the midbody of dividing cells during later stages of cytokinesis (Supplementary Fig. 1B). These data suggested a potential role for G2L1 in cell division.

### G2L1 depletion results in aberrant cell division leading to nuclear abnormalities

**Figure 3:**
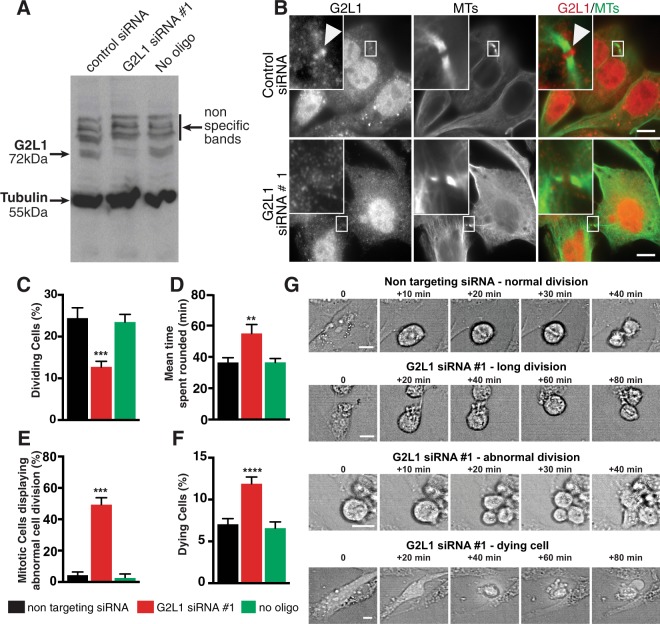
G2L1 knock down results in abnormal cell division. (**A**) Western blot showing knock down of G2L1 in U2OS cells. (B) Panel represents U2OS cells transfected with siRNA control and G2L1 siRNA ♯ 1. Cells were fixed and stained for endogenous G2L1 (red) and MTs (green). White boxes in the top left corners of images show the midbody of dividing cells and magnifications. White arrowheads indicate accumulation of G2L1 at the midbody of control cells. Graph represents quantification of (**C**) percentage of dividing cells, (**D**) time when cells are rounded, (**E**) abnormal cell division (%), (**F**) cell death, in control and G2L1 knock down samples. Data presented are mean values (±s.e.m.) of 3 independent experiments, black asterisks indicate ***P < 0.001, One-way ANOVA with Sidak post-hoc test. (**G**) Representative still-frame images of U2OS cells after either non targeting siRNA or G2L1-specific siRNA taken from live-cell time-lapse movies used to quantify mitotic cell division phenotypes. Scale bar, 10 µm.

**Figure 4:**
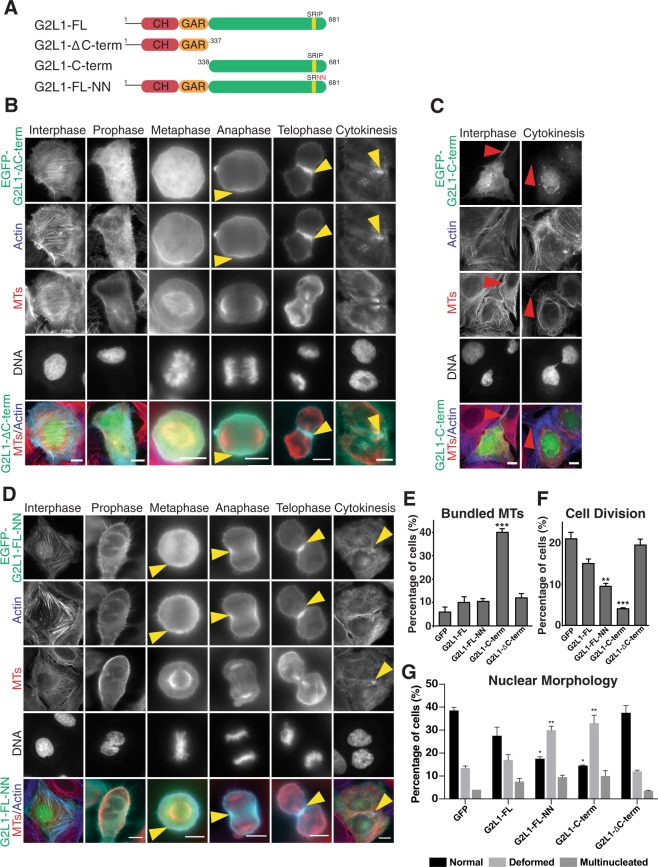
G2L1 mutants show different localisation during cell division and influence nuclear morphology and cell division rate. (**A**) Schematic representation of GAS2L1 constructs used in this study. The calponin homology (CH) and GAS2-related (GAR) domains are depicted in red and orange, respectively, and the number of amino acids for each construct is noted above the C-termini (green). The IP motives responsible for end-binding proteins (EB) binding are denoted above their respective C-termini, SxIP motifs are yellow, S/TxLP motifs are red. Note that schematic represents the beta isoforms of G2L1. Panel of images representing U2OS cells expressing EGFP-tagged (**B**) G2L1-ΔC-term, (**C**) G2L1-C-term and (**D**) G2L1-FL-NN stained for MTs (red), actin (blue) and DNA in different phases of the cell division. Arrowheads show accumulation of indicated G2L1 mutant; yellow at actin-related and red at MT-related mitotic structures in dividing cells. Scale bars indicate 10 µm. Graphs showing quantification of (**E**) cells displaying bundled MTs, (**F**) cells undergoing division and (**G**) changes in nuclear morphology in cells expressing indicated G2L1 constructs. Data presented are mean values (±s.e.m) of 3 independent experiments (n > 60 cells), black asterisks indicate *P < 0.01, **P < 0.005, compared to G2L1-FL-GFP, one-way ANOVA with Sidak post-hoc test.

To determine the potential role of G2L1 in cell division we depleted the endogenous protein using three different G2L1-specific siRNA. We confirmed siRNA knock down of G2L1 by western blotting (Fig. 3A) and by quantitative PCR (qPCR), which showed a 60–80% reduction of G2L1 mRNA levels compared to control cells (Supplementary Fig. 1C). To visually assess G2L1 knock down in cells, we expressed empty GFP-vector together with siRNA oligos and stained these cells for endogenous G2L1. As endogenous G2L1 was primarily detectable in cells at its accumulation at the midbody during the later stages of cytokinesis, we analysed localisation of endogenous G2L1 at constriction zones. The control cells expressing empty GFP-vector together with control siRNA were positive for G2L1 at the midbody. However, none of the cells expressing empty GFP-vector together with G2L1-specific siRNA showed localisation of G2L1 at the constriction sites of the midbody (Fig. 3B and Supplementary Fig. 1D). Co-staining with DAPI further revealed that of those cells depleted of G2L1, a large population (~60%) displayed either deformed nuclei or were multinucleated (Supplementary Fig. 1E). The population of cells that had undergone G2L1 knock down also exhibited (1) a significantly reduced number of cells undergoing division (60%) compared to control cells (Fig. 3C), (2) significantly increased length of time staying rounded during mitosis (Fig. 3D), (3) more frequent abnormal cell division (Fig. 3E), and (4) increased cell death (Fig. 3F). Examples of these phenotypes are shown in Fig. 3G. These findings suggest that G2L1 plays an important role in cell division.

### Expression of the C-terminal tail of G2L1 blocks cell division

To gain further mechanistic insight into the role of G2L1 in the regulation of cell division we expressed mutated forms of G2L1 in U2OS cells and compared them with the full-length form (G2L1-FL) (Fig. 4A). We first assessed whether expression of a G2L1 construct deficient in actin or MT binding had an effect on cell division. To achieve this we expressed G2L1 either lacking the whole MT binding C-terminal tail (G2L1-ΔC-term) or consisting only of the C-terminal tail (G2L1-C-term), which binds MTs but not actin. During mitosis, G2L1-ΔC-term localised predominantly to actin structures including the contractile ring and the cleavage furrow (Fig. 4B). The expression of this construct had no effect on cell division (Fig. 4B,F). Surprisingly, and in stark contrast to cells expressing G2L1-FL or G2L1-ΔC-term, expression of the C-terminal tail only, which also triggered MT bundling (Fig. 4C,E), inhibited cell division by 80% (Fig. 4F), a rate exceeding that seen after G2L1 knock down. Expression of this construct also led to a large number of cells showing nuclear deformation and multinucleation (Fig. 4C,G). These effects were predominantly observed in cells with higher expression levels of G2L1-C-term. Previously the expression level of G2L1-C-term has been shown to affect the protein’s behaviour: higher expression levels resulted in MT stabilisation while lower levels allowed MT tip-tracking^13^.

### Binding of G2L1 to EB-proteins is essential for normal cell division

The association of EB protein with G2L1 is essential for the ability of G2L1 to mediate cross-communication between actin and MTs^13^. EB proteins are also known to be essential for coordinated cell division^20^. To examine a potential link between EB binding and G2L1 function in cell division we transfected U2OS cells with a G2L1 mutant deficient in EB binding (G2L1-FL-NN). In comparison to wild-type, G2L1-FL-NN showed a more diffuse localisation at mitotic structures suggesting diminished binding strength. G2L1-FL-NN localised mainly to the contractile ring and cleavage furrow during later stages of mitosis. It still localised consistently, albeit weakly, to centrosomes, to the mitotic spindle and to constriction zones of the midbody (Fig. 4D). Despite its localisation to mitotic structures, G2L1-FL-NN overexpression essentially phenocopied the cell division phenotypes observed in cells depleted of G2L1; an increased number of multinucleated cells and cells with nuclear deformation (Fig. 4D,G), and an overall reduction of the number of cells undergoing cell division (50%) (Fig. 4F). Taken together, these data demonstrate that the binding of G2L1 to both MTs and to EB-proteins is essential for correct nuclear morphology and successful cell division. Our results suggest that G2L1 is a critical regulator of cell division, and that its function during mitosis is dependent on its binding to EB proteins.

## Discussion

Here, we show that G2L1 depletion results in multinucleation, nuclear deformation and an overall reduction in the rate of cell division. Using G2L1 mutants we show that the binding of the C-terminus to EB proteins has a critical role in the observed processes. Results showing that the expression of a point mutant deficient in binding to EB proteins mimics in part the defects seen in G2L1 depleted cells show that the presence of G2L1, as well as its capability to bind EB proteins, is vital for coordinated regulation of cell division.

### The role of GAS2 family members in the cell cycle

A recent study has described an important function in G2L1 for attaching microtubules and actin to centrosomes and in mediating centrosome splitting/disjunction at the beginning of mitosis, where siRNA-mediated knock-down of G2L1 prevented centrosome separation^1^. Here, we also observed localisation of G2L1 (endogenous and GFP-expressed) to centrosomes (Supplementary Fig. 1B, Supplemental Movie 1), which could account for the reduced number of cells entering mitosis (Fig. 3C) and the defects in cytokinesis (Fig. 3D–F). Interestingly, Au *et al*.^1^ used a similar, but not identical, G2L1 C-terminal overexpression construct as we used in the present study. In their study, the construct acted as a dominant negative construct, preventing G2L1 recruitment to centrosomes^1^. Although we have not tested whether the defects in cell division we observe are a consequence of our C-terminal construct replacing wild-type G2L1 from centrosomes and the midbody (therefore acting as a dominant negative construct), our overexpression data of G2L1-C-term suggest this to be the case (Fig. 4F): G2L1-C-term expression bundled microtubules (Fig. 4E), and resulted in a similar phenotype (reduced cell division, Fig. 4F; disrupted nuclear morphology, Fig. 4G) as G2L1 depletion. Taken together, our results and those of Au *et al*.^1^ suggest that disrupting centrosomal disjunction has further downstream effects on cell division.

Some of our observations also share similarities with those observed for G2L3^16,17^. Both G2L1 and G2L3 localise to mitotic structures such as the spindle, the cleavage furrow and the midbody, and both are required for the completion of cytokinesis. However, there are also essential differences between them. Firstly, in contrast to G2L1, the overexpression of G2L3 blocks cell division^16,17^, (our unpublished observation). Secondly, the effect of G2L1 seems strongly dependent on EB protein binding via SxIP motifs that are absent in G2L3. The observation of others that G2L3 is dramatically upregulated during G2/M phase whilst G2L1 is expressed throughout the cell cycle at low levels^16^, underscores a likely different role of the two proteins during mitosis.

What are the specific functions of the two proteins? G2L3 was speculated to have a role in regulating the components of the contractile ring, since depletion of Anillin, mDia2 or also the kinesin MCAK resulted in a similar myosin II-dependent oscillatory phenotype due to problems in cell separation^25–29^. While some of these functions may be shared by G2L3 and G2L1, the role of G2L1 seems to be strictly associated with EB-protein function during mitosis.

### The potential role of G2L1 and EB proteins in cell division

Efficient G2L1-mediated crosstalk between actin and MTs depends on its ability to interact with EB proteins^13^. Here, we provide evidence that this interaction is also crucial for cell cycle progression. As essential regulators of MT dynamics it may not be surprising that EB proteins are also implicated in the regulation of mitosis^19^. Of the three mammalian EB proteins, EB1 and EB3 in particular have been linked to mitotic events^30^. The loss of EB1, for example, can disrupt stable positioning of the mitotic spindle and normal alignment of chromosomes, leading to chromosome missegregation^31^. This could occur downstream of the centrosome separation phenotype observed after siRNA-mediated knock down of G2L1^1^. Disrupted spindle positioning and chromosome alignment could explain why cells depleted of G2L1 were rounded for longer prior to division (Fig. 3D). Furthermore, chromosome missegregation could explain the abnormal cell division and nuclear morphology observed after siRNA-mediated knock down of G2L1 (Fig. 3E). Our results suggest that the interaction between G2L1 and EB1 is particularly important for this process, since expressing a G2L1 construct with point mutations in the SxIP motif gave a similar phenotype as G2L1 siRNA-mediated knock down (Fig. 4F,G). These findings are also in line with a previous study that showed a role for the interaction between the Ser/Thr-X-Ile/Leu-P (SxIP) motif of the EB1-binding protein APC (adenomatous polyposis coli protein) and EB1 in chromatin deformation^32^.

Depletion of EB3 seems to have an even more dramatic effect in that it can block the progression of cell division^3^. A more recent publication outlined that EB1 and EB3 have temporally distinct roles during cell division^19^. Whereas EB1 had a role in spindle positioning, EB3 was required for the efficient link between astral microtubules and cortical actin. Moreover, EB3 was important for the stabilisation of FAs required for daughter cell spreading, and it mediated midbody stabilisation necessary for efficient cytokinesis^19^. Since G2L1 binds EB1 and EB3^13^ it is possible that it is involved in the regulation of both EB proteins. An essential regulatory role of G2L1 association with EB proteins was recently observed; expression of G2L1 constructs deficient in EB-protein binding, in contrast to its wild-type counterparts, does not promote centrosome separation. Our observations showing that expression of the EB protein-binding mutants display significant changes in nuclear morphology frequently associated with multinucleation shows a critical role for G2L1-EB association in cytokinesis. This could occur through a similar mechanism as observed after knock down of G2L3^16^. Further experiments will be required to determine the precise role of the intimate relationship between G2L proteins and EB proteins in time and space during the different phases of mitosis.

### Hypothetical model of G2L1 function during cell division

**Figure 5:**
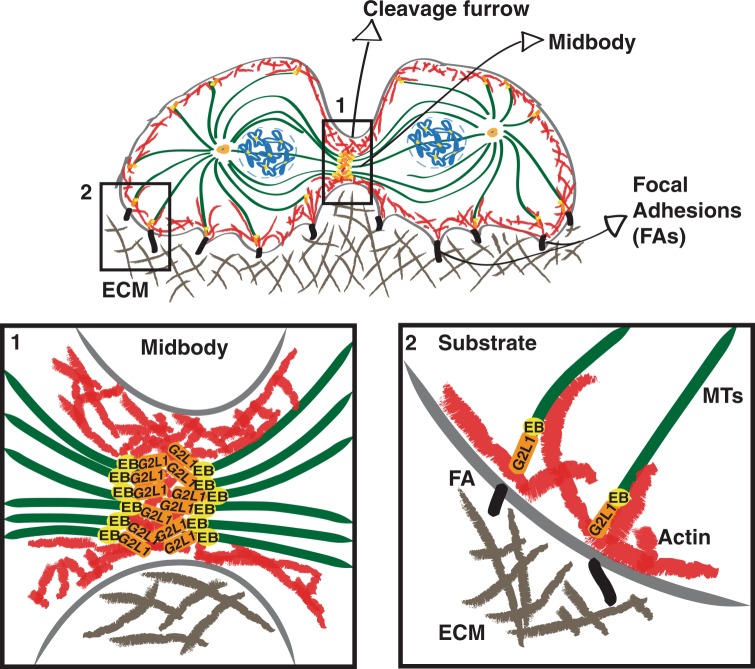
Proposed model of G2L1 role in EB-mediated actin-MT crosscommunication during cytokinesis. During cell division G2L1 mediates actin-MT crosstalk through its interactions with EB proteins. (1) At the midbody G2L1 seems to be involved in the crosstalk between actin at the cleavage furrow and MTs at the midbody through its interactions with EB proteins. Coordinated regulation of these structures allows successful separation of daughter cells during later stages of cytokinesis. (2) In close proximity of the cell anchorage to the ECM through FAs, G2L1 seems to play a role in EB-mediated attachment of astral MTs to actin cortex. It allows coordinated spreading of daughter cells, which is required for successful completion of cytokinesis.

Overall, our data support a hypothetical model whereby G2L1 coordinates the interactions of astral MTs with the cortical actin network in an EB-protein dependent manner (Fig. 5). In this model G2L1 links MT structures of the midbody via EB proteins to the contractile cortical actomyosin machinery whose forces are required for separating the two daughter cells during cytokinesis. G2L1 may also be important for tethering astral microtubules to the actin cortex, which is required for mitotic spindle stabilization and positioning as well as for the anchoring of daughter cells to the ECM, which is necessary for FA formation and daughter cell spreading.

## Materials and Methods

### Cell Culture and Transfections

U2OS cells were cultured in DMEM (Sigma Aldrich, Dorset, UK) supplemented with 10% FBS, and 1% glutamine and 1% non-essential amino acids in a 5% CO_2_ humidified incubator. For transfections cells were plated in 6-well dishes (Corning Incorporated, USA) and transfected using 0.3–2 μg of DNA and Lipofectamine 2000 (Invitrogen, Paisley, UK) in accordance with manufacturer’s instructions. For immunofluorescence, cells were replated after 3–4 hours in glass-bottomed dishes (MatTek Corporation, Ashland, USA) coated with 10 μg/ml bovine fibronectin (pFN; Sigma) and incubated for 24 h in a 5% CO_2_ humidified incubator.

### siRNA transfection

For G2L1 knock down the following GAS2L1-specific siRNAs (Sigma-Aldrich, UK) were used:

siRNA#1: NM_006478, SASI_Hs01_00153525

siRNA#2: NM_152236, SASI_Hs01_00213413

siRNA#3: NM_152237, SASI_Hs01_00365736

and a combination of all three siRNA#1–3.

G2L1-specific siRNAs and siRNA control as well as a sample with no siRNA added (no oligo) were transfected to U2OS cells (10 cm cell culture dish) at concentrations of 10 nM using Lipofectamine 2000 (Life Technologies, UK), according to the manufacturer’s protocol. Transfection reactions were performed in serum-free DMEM (Sigma-Aldrich, UK). Cells were incubated overnight in a 5% CO_2_ humidified incubator; the medium was then changed to DMEM-containing serum and incubated for a further 24, 48 and 72 hours. A second round of siRNA transfection was performed 48 hours after the first round.

### RT-qPCR

Total RNA was extracted from U2OS cells using the ReliaPrep RNA Cell Miniprep System (Promega, UK), 48 hours post-transfection. 0.5 μg total RNA was used for cDNA synthesis with MMLV reverse transcriptase and either random hexamers or oligo(dT) primers together with control RNA template according to manufacturer manual (Tetro cDNA Synthesis Kit, Bioline, UK). RT-qPCR was performed using SYBR Green Mastermix (GoTaq 1-Step RT-qPCR, Promega, UK). Primers for G2L1 were designed using the NCBI primer-BLAST oligos designer web tool.

Primer sequences are:

Forward primer: CTC ATC TTT GTG CGG GTG CT

Reverse primer: AGG TAA TGC TCC AGC GTG TC

The efficiency of each primer set for RT-qPCR was determined to be between 95 and 100%. Primers were purchased from Life Technologies (UK). Real-time qPCR analysis was carried out using CFX96/384 instruments (Bio-Rad) and the GoTaq qPCR Mastermix Kit (Promega). Expression analysis was performed in triplicate using CFX Manager software v3.0 (Bio-Rad), with samples normalised to a combination of TATA box binding protein (TBP) and glyceraldehyde 3-phosphate dehydrogenase (GAPDH) expression. Gene expression data (relative to control cell expression) from across replicate experiments was entered directly into Microsoft Excel, where it was used for analysis using One-way ANOVA.

### Western Blotting

To test detection of G2L1 we used a commercially available G2L1 antibody (Abgent). WB experiments were performed on non-transfected and G2L1-FL-GFP transfected U2OS cells collected from 6-well plates. WB experiments used to analyse efficiency of G2L1 siRNA knock down were performed on siRNA control, G2L1#1 and no oligo transfected U2OS cells collected from 10 cm cell culture dishes. Cells were washed once with cold PBS (Lonza, UK) and scraped into 200/500 μl 2× Laemmli buffer. Samples were homogenized by 3 passes through a syringe with a 25-gauge needle followed by centrifugation at 21,000 *g* for 15 min at 4 °C. Next, samples were boiled at 95 °C for 5 min. Samples were resolved by 7.5% polyacrylamide gel during SDS-PAGE electrophoresis (120 V–1 h) and transferred on nitrocellulose membrane during WB transfer (250 mA–2.5 h). After transfer the membrane was blocked with 5% milk for 1 h at RT, washed 3x in PBS + 0.1% Tween 20, and incubated with the primary rabbit anti-human G2L1 antibody (Abgent) at 1:1000 dilution and mouse anti-tubulin (DM1A, Sigma-Aldrich, UK) antibody, at 1:5000 dilution in 3% milk + 0.1% Tween 20 (incubated o/n at 4 °C). After incubation with the primary antibodies, the membrane was washed 3x with PBS + 0.1% Tween 20 and then incubated with the secondary antibody:, goat anti-rabbit and goat anti-mouse suitable for the Odyssey system, were used in 1:5000 dilution/goat anti-rabbit-HRP conjugate (Bio-Rad) and goat anti-mouse HRP conjugate (Bio-Rad) suitable for the Enhanced Chemiluminescence (ECL) system in 1:5000 dilution, in 3% milk + 0.1% Tween 20 and incubated for 1 h at RT in 3% milk + 0.1% Tween 20 and incubated for 1 h at RT.

### Antibodies and immunofluorescence imaging

Cells were fixed and permeabilised with 3% paraformaldehyde (Sigma-Aldrich, UK) containing 0.25% Triton X-100 (Sigma-Aldrich, UK) and 0.05% glutaraldehyde (Sigma-Aldrich, UK) for 15 mins, before being washed in PBS (Lonza, UK). Cells were incubated with anti-tubulin (DM1A, Sigma-Aldrich, UK) antibody, at 1:500 dilution, and anti-G2L1 (Abgent) 1:100 dilution, followed by DyLight 488, 594, 649-conjugated secondary antibodies (Jackson ImmunoResearch Laboratories, Suffolk, UK). For actin, Texas Red, FITC or Alexa Fluor 633-labelled phalloidin (Life Technologies, UK) was added together with the secondary antibody, at 1:250 dilution. For nuclei staining cells were incubated with DAPI at 1:100 dilution for 10 min to detect DNA. For the EB1 staining (1A11/4, Santa Cruz), cells were fixed in −20 °C methanol for 5 min, before being rehydrated in PBS and stained using anti-tubulin antibodies (as above). Cells were then imaged using an oil-immersed 100X objective, with 1.35 numerical aperture on an inverted microscope (IX71; Olympus) controlled by a Deltavision system (Applied Precision, Washington, USA). Images were captured using a Coolsnap HQ CCD camera (Princeton Instruments, Lurgan, UK).

### Live-cell imaging

For live-imaging experiments U2OS cells were transfected with G2L1-GFP, together with mCherry-tubulin. After transfection cells were replated on fibronectin-coated glass-bottom dishes and spread for 24 h prior to live imaging. Cells were imaged in Ham’s F12 medium supplemented with 25 mM HEPES, 1% L-glutamine, 1% penicillin/streptomycin with pH adjusted to 7.3 with NaOH (as described in ref.^33^). A CSU-X1 spinning disk (Yokagowa) confocal microscope (Zeiss Axio-Observer Z1) equipped with a 60x/1.40 oil Plan-Apochromat objective, Evolve EMCCD camera (Photometrics) and motorised XYZ stage (AIS) was used to acquire images every 10 minutes. The 488 nm and 561 nm lasers were controlled using an Acousto-optical tunable filter through a laserstack (Intelligent Imaging Innovations (3i)).

### Image Processing

We specifically analysed cells that expressed the various constructs (control vs. G2L1 constructs) at very similar levels (assessed by fluorescence intensity), enabling us to make valid comparisons between conditions.

To measure colocalisation of GAS2 family members and EB proteins, RGB merged images were prepared using Fiji. MATLAB was used to create fluorescence intensity line profiles over merged images and graphs representing normalised fluorescence intensity over the drawn line.

To analyse changes in nuclear morphology and cell division rate of cells transfected with different constructs of G2L1, cells were stained for DNA using DAPI and 60 randomly selected cells were manually screened.

Adobe Illustrator CS4 and Fiji Image J were used in the preparation of figures for this manuscript.

### Statistical analysis

Student’s t-test and Anova statistical analysis was performed using Microsoft Excel or GraphPad Prism to compare cell division, nuclear morphology and siRNA knock down efficiency.

## Supplementary information

Supplementary Information

Supplemental Movie 1
