# Supplementary material for: GAS2-like 1 coordinates cell division through its association with end-binding proteins
Source: Sci Rep. 2019 Apr 9;9:5805. doi: 10.1038/s41598-019-42242-6 (PMC6456587; doi:10.1038/s41598-019-42242-6)
Supplement: Supplementary file 1 — Supplementary Information [file 41598_2019_42242_MOESM1_ESM.pdf]

GAS2-like 1 coordinates cell division through its association with end-binding proteins

Keywords: GAS2 family, GAS2-like 1, end-binding protein (EB), microtubules, actin, cell division

Running title: G2L1 coordinates cell division via EBs

Alicja Nazgiewicz, Paul Atherton and Christoph Ballestrem*

Wellcome Trust Centre for Cell-Matrix Research, Faculty of Biology, Medicine and Health, University of Manchester, M13 9PT, UK.

Correspondence should be addressed to Christoph Ballestrem

Email: christoph.ballestrem@manchester.ac.uk

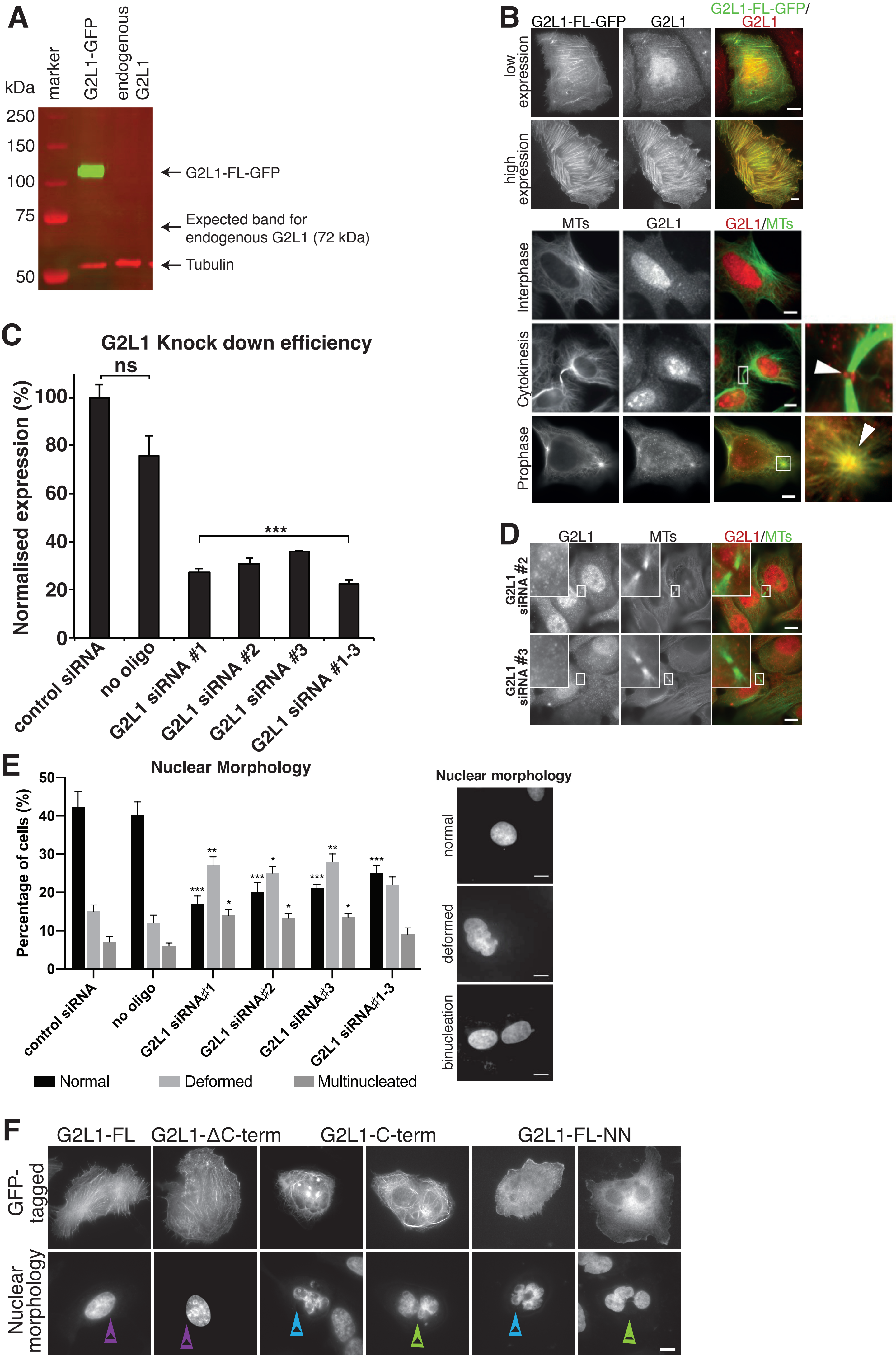

A
marker
G2L1-GFP
endogenous G2L1
kDa
250
150
100
75
50
G2L1-FL-GFP
Expected band for endogenous G2L1 (72 kDa)
Tubulin
B
G2L1-FL-GFP
G2L1
G2L1-FL-GFP/G2L1
low expression
high expression
MTs
G2L1
G2L1/MTs
Interphase
Cytokinesis
Prophase
C
G2L1 Knock down efficiency
ns
***
Normalised expression (%)
100
80
60
40
20
0
control siRNA
no oligo
G2L1 siRNA #1
G2L1 siRNA #2
G2L1 siRNA #3
G2L1 siRNA #1-3
D
G2L1
MTs
G2L1/MTs
G2L1 siRNA #2
G2L1 siRNA #3
E
Nuclear Morphology
Percentage of cells (%)
50
40
30
20
10
0
control siRNA
no oligo
G2L1 siRNA#1
G2L1 siRNA#2
G2L1 siRNA#3
G2L1 siRNA#1-3
Normal
Deformed
Multinucleated
Nuclear morphology
normal
deformed
binucleation
F
G2L1-FL
G2L1-ΔC-term
G2L1-C-term
G2L1-FL-NN
GFP-tagged
Nuclear morphology

Supplementary Figure 1.

(A) WB of U2OS cell extracts using anti-G2L1 Ab (green) and anti-tubulin Ab (red) and the Odyssey imaging system to show expression levels of the corresponding proteins. (**B**) U2-OS cells expressing G2L1-FL-GFP (green) at different levels, stained for endogenous G2L1 (red). Lower panel: U2OS cells stained for MTs (green) and G2L1 (red) during interphase, cytokinesis, or prophase. White arrowhead indicates accumulation of endogenous G2L1 at the constriction sites of the midbody during later stages of cytokinesis, and endogenous G2L1 at the centrosome during prophase. (**C**) Graph showing normalised expression of G2L1 after knock down using three different oligos (siRNA ♯1, ♯2 and ♯3), measured by RT-qPCR. *** indicates $p<0.001$, one-way ANOVA, Sidak post-hoc test. Data are pooled from 3 independent experiments. (**D**) Panel showing U2OS cells transfected with G2L1 siRNA ♯ 2 and ♯ 3. Cells were fixed and stained for endogenous G2L1 (red) and MTs (green). White boxes in the top left corners show magnified images of the midbody of dividing cells. (**E**) Quantification and representative images of U2OS cells showing normal or deformed nuclei, or binucleation after G2L1-specific or control-siRNA knock-down. * indicates $p<0.05$, ** $p<0.01$, *** $p<0.001$, one-way ANOVA with Sidak post-hoc test. Results are pooled from three independent repeats. **(F**) Panel of images showing U2OS cells expressing the indicated G2L1 construct and their nuclear morphology; normal (purple), deformed (blue) and multinucleated (green). Scale bars, 10 μm.

Supplemental Movie 1. G2L1 during cell division.

Spinning disk confocal movie of a U2OS cells expressing GFP-G2L1-FL (magenta) and mCherry-Tubulin (green) undergoing cell division. Note the localization of G2L1 to the midbody during cytokinesis. Note also the localisation of G2L1 to the centrosomes before and after division. Images were acquired every 10 minutes for 16 hours. Scale bar indicates 10 μm.
